# Stability of Mina v2 for Robot-Assisted Balance and Locomotion

**DOI:** 10.3389/fnbot.2018.00062

**Published:** 2018-10-15

**Authors:** Carlotta Mummolo, William Z. Peng, Shlok Agarwal, Robert Griffin, Peter D. Neuhaus, Joo H. Kim

**Affiliations:** ^1^Department of Mechanical and Aerospace Engineering, New York University, Brooklyn, NY, United States; ^2^Florida Institute for Human and Machine Cognition, Pensacola, FL, United States

**Keywords:** robotic exoskeleton, balance stability boundary, combined human-exoskeleton system, linear linkage actuator, Mina v2

## Abstract

The assessment of the risk of falling during robot-assisted locomotion is critical for gait control and operator safety, but has not yet been addressed through a systematic and quantitative approach. In this study, the balance stability of Mina v2, a recently developed powered lower-limb robotic exoskeleton, is evaluated using an algorithmic framework based on center of mass (COM)- and joint-space dynamics. The equivalent mechanical model of the combined human-exoskeleton system in the sagittal plane is established and used for balance stability analysis. The properties of the Linear Linkage Actuator, which is custom-designed for Mina v2, are analyzed to obtain mathematical models of torque-velocity limits, and are implemented as constraint functions in the optimization formulation. For given feet configurations of the robotic exoskeleton during flat ground walking, the algorithm evaluates the maximum allowable COM velocity perturbations along the fore-aft directions at each COM position of the system. The resulting velocity extrema form the contact-specific balance stability boundaries (BSBs) of the combined system in the COM state space, which represent the thresholds between balanced and unbalanced states for given contact configurations. The BSBs are obtained for the operation of Mina v2 without crutches, thus quantifying Mina v2's capability of maintaining balance through the support of the leg(s). Stability boundaries in single and double leg supports are used to analyze the robot's stability performance during flat ground walking experiments, and provide design and control implications for future development of crutch-less robotic exoskeletons.

## Introduction

Robotic exoskeletons have the potential to change the day-to-day life of countless individuals with mobility impairment. Commercial lower-limb exoskeletons, such as ReWalk (Esquenazi et al., 2012), Ekso (Ekso, 2018), and Indego (Parker, 2018) have made significant progress in restoring the mobility of individuals with spinal cord injury (SCI) (e.g., paraplegics or paraparetics). Current research addresses various aspects of exoskeleton functionality, such as providing mobility to patients who are confined to a wheelchair (Esquenazi et al., 2012) or are suffering from muscular weakness (e.g., the elderly and infirm; Sankai, 2010), improving rehabilitation (neurological or orthopedic) and recovery efficacy (Colombo et al., 2000; Veneman et al., 2007), and augmenting the performance of healthy individuals during heavy load carrying tasks (Guizzo and Goldstein, 2005; Walsh et al., 2007). Recent achievements in lower-limb exoskeleton assistance include successes in robot-assisted walking (Raj et al., 2011; Hassan et al., 2014; Sanz-Merodio et al., 2014; Griffin et al., 2017), stair ascent and descent (Xu et al., 2017), and sit-to-stand movements (Tsukahara et al., 2010). Additionally, other studies have focused on the reduction of exoskeleton's energy consumption during task performance through the use of elastic and dissipative elements (Wang et al., 2011; Kim et al., 2015).

In walking applications, the goal is to achieve stable robot-assisted locomotion that is adaptable to various terrain and gait parameters, and at an increased range of speeds and larger step lengths, for which ankle actuation is essential. The robotic exoskeleton H2 was the first robotic exoskeleton for gait rehabilitation to include ankle actuation, and has been followed by the development of other designs employing active ankles (Bortole et al., 2015). The recently developed assistive device Mina v2 (Griffin et al., 2017) includes ankle, hip, and knee actuations with the intention of achieving more human-like lower-limb motion during gait. Its powered plantar flexion allows the human operator to navigate various environments, such as stairs and ramps, as demonstrated during the 2016 Cybathlon competition, and to reliably achieve a conservative walking speed of 0.29 m/s (Griffin et al., 2017). These recent advancements could progress toward robot-assisted gait that requires reduced effort from the user (Griffin et al., 2017) and has desired dynamic walking characteristics, e.g., similar to normal or load-carrying human walking (Mummolo and Kim, 2013; Mummolo et al., 2013, 2016). The effort of translating human locomotion principles into robotic solutions requires quantitative benchmarks to evaluate the human-like performance of robotic assistive devices (Neuhaus et al., 2011). In existing studies, analyses of robot-assisted gait have been conducted with data collected from a sensorimotor wearable robotic system (Raj et al., 2011), a versatile instrumented cane together with body worn sensors (Hassan et al., 2014; Lancini et al., 2016), and motor encoders (Griffin et al., 2017). Given these data, several outcomes can be used to benchmark the exoskeleton-assisted gait of SCI individuals against normal gait (Torricelli et al., 2015).

While human-like dynamic walking is a desired performance goal in the design of exoskeletons for robot-assisted locomotion (Barbareschi et al., 2015; Li et al., 2015; Agrawal et al., 2017), user safety remains the primary concern. In addition to employing a structural design that guarantees the physical safety of human-robot interactions (i.e., the user should not experience physical discomfort or injury by wearing and operating the robot), proper control design must also be implemented to stabilize the system so that the user is also protected from the risk of injury due to falls. To guarantee stable robot-assisted movements, a systematic and quantitative analysis of the balance stability of the human operator wearing the exoskeleton suit is required from its initial mechanical design to its final assessment. Currently, maintaining balance during robot-assisted gait remains a challenging problem and the operator often relies on the support of additional devices to improve balance. For example, one study (Slavnic et al., 2010) considered the use of a powered exoskeleton integrated with a wheeled mobile platform to provide balance during walking. In several cases, the operator relies on crutches or walkers in order to maintain balance (Acosta-Marquez and Bradley, 2005; Strausser and Kazerooni, 2011; Esquenazi et al., 2012; Farris et al., 2014; Stücheli et al., 2017). While real-time gait planning strategies using crutches as balancing aids have been implemented to produce stable and natural walking (Zhang et al., 2015a,b), they are far from ideal solutions. The use of crutches is often incompatible with the surrounding environment, restricts the operator's use of hands, limits the achievable walking speed, and requires a significant amount of upper limb strength during walking and standing, fatiguing the user (Griffin et al., 2017).

Researchers have recently begun to address the balance stability analysis for humans wearing exoskeletons in the absence of crutches. The design of a hybrid drive exoskeleton has been proposed (Hyon et al., 2011, 2013), in which a combination of pneumatic muscles and electric motors are used to provide sufficient torque and controllability in order to balance without crutches. In those studies, the analysis was focused on the robotic system alone, excluding its human component, and was based on a limited performance evaluation and validation. The design of a robotic exoskeleton with a balance stabilizer mechanism has been proposed and tested for use on SCI subjects (Li et al., 2015), which requires further improvement in order to manage significant shifts in body weight in the coronal plane. Control methods have been developed to provide active gait assistance in both sagittal and frontal planes (Wang et al., 2013, 2015) for the MINDWALKER exoskeleton, and its stable walking without crutches has been demonstrated for healthy subjects (but not yet for SCI paraplegics; Wang et al., 2015). Human and robot balance stability criteria, for instance, based on the capture point and extrapolated center of mass concepts, have also served as promising sources of inspiration for robot-assisted balance control (Huynh et al., 2016; Zhang et al., 2018) and balance recovery against slipping-like perturbation (Monaco et al., 2017); these studies have addressed healthy subjects or subjects with significant voluntary abilities retained. Very recently, ankle joints powered via variable stiffness actuators, which mimic the modulation of muscle impedance in the human ankle for balancing, were proposed to replace constant stiffness actuators (Ugurlu et al., 2016) in order to provide more favorable external disturbance dissipation. However, depending on the disturbance amplitude, the desired ankle stiffness may not be physically realizable with the variable stiffness actuator, and the system can still fail to maintain balance. In the absence of comprehensive human-exoskeleton combined models and a systematic balance stability analysis of lower-limb exoskeletons, control strategies will continue to rely heavily on additional balancing aids, such as crutches and *ad hoc* criteria.

There is no commonly applicable and comprehensive framework for the balance stability analysis of robot-assisted locomotion so far. The difficulty arises in part from the traditional challenges in determining balancing vs. falling conditions for general legged systems (Mummolo et al., 2017), but also from the modeling complexity of actuator and multibody dynamics of the combined human-exoskeleton system. When addressing the balance stability of robot-assisted gaits, it is essential to establish an accurate model that can describe the dynamics of the combined system representing the human operator wearing the robotic exoskeleton. While several models exist to describe the multibody dynamics of human and robotic biped systems separately, few studies take into account the combined system's dynamics. One study that proposed a hybrid zero dynamics controller for robot-assisted gait treated the human lower body and the exoskeleton as a lumped rigid-body system due to the lack of actuation from the legs (Agrawal et al., 2017). Another study used an improved human-exoskeleton model that introduced compliance at each joint by adding spring-mass-damper systems with parameters obtained through optimization using data from push recovery experiments (Schemschat et al., 2016).

In this study, the balance and locomotion stability characteristics of Mina v2 are systematically evaluated for its typical foot-ground contact configurations. An equivalent model representing the human-exoskeleton system dynamics is established, where the mechanical and actuation models of the human body and the robotic device are combined. A center of mass (COM)-state-based criterion is used to characterize the set of balanced states of the combined human-exoskeleton system in legged support without crutches. For a given foot-ground contact configuration of the combined system, the criterion determines the threshold between balanced and unbalanced states of the system with respect to that configuration. The balance stability criterion is applied to the equivalent model in single and double contact configurations to quantify the capability of Mina v2 to maintain balance through the support of the leg(s), for instance, during swing and transfer gait phases, respectively. The application of the balance stability criterion is demonstrated by using experimental data to characterize the state of balance of robot-assisted walking motions.

## Robotic exoskeleton Mina v2

**Figure 1:**
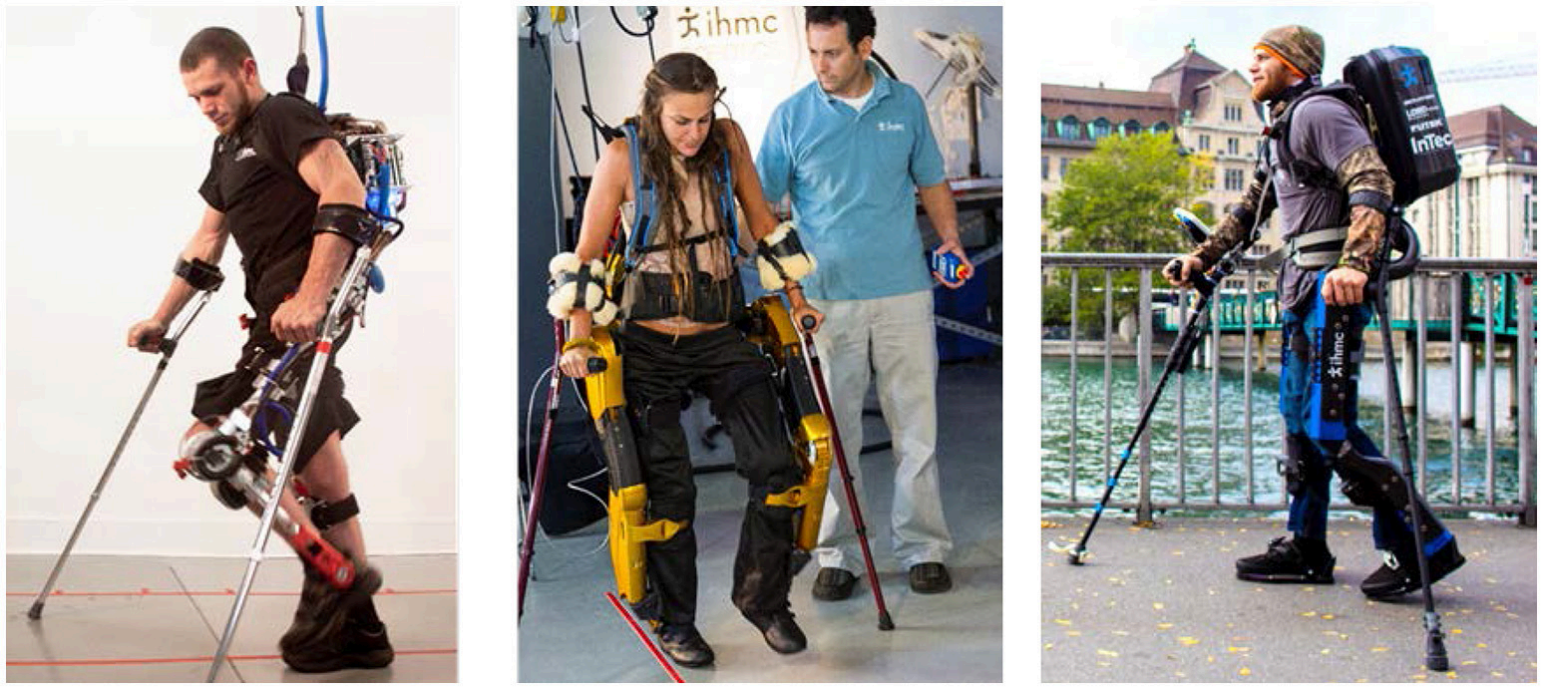
Mina v1 (Left), X1 (Center), and Mina v2 (Right) exoskeletons (permission for image reproduction granted by the participants).

Mina v2 exoskeleton is a prototype paraplegic mobility assistance device designed and built by the authors at the Florida Institute for Human and Machine Cognition (IHMC), and is the third in a series of devices designed to provide upright mobility for people with lower extremity paralysis (Figure 1). Each of these devices provides sagittal plane motion of the legs while its upright balance is provided by the user with required forearm crutches (i.e., no balance controller currently implemented). These wearable devices rigidly constrain the operator's joint position and track a commanded joint profile from the walking controller.

The exoskeleton Mina v1 (Kwa et al., 2009; Figure 1) had four sagittal plane motors, at the hips and knees, and two passive compliant ankle joints. The actuators were brushless motors with a harmonic drive gear reduction and could be used with either stiff position control or torque control (Neuhaus et al., 2011). Similar to Mina v1, the exoskeleton X1 (Figure 1) had four sagittal plane motors, at the hips and knees, and passive compliant ankle joints. Driving each powered joint was a series elastic actuator that could allow for position or force control.

Mina v2 (Figure 1) has actuators at the hips and knees like its predecessors, and, in addition, includes an actuator for each ankle joint, resulting in full actuation in the sagittal plane. The powered ankle plantar flexion and dorsiflexion provide the exoskeleton system with stability and mobility, and is motivated by the analysis of human walking (Winter, 1990), which shows that the ankle plays an important role by injecting energy during the toe-off (terminal stance) phase of the trailing stance foot and allowing for dynamic walking (Torricelli et al., 2016). Moreover, the modulation of ankle torque can control the center of pressure displacement within the contact area during mid stance (Perry and Burnfield, 1992), which is a well-known fundamental strategy for balance control.

### Joint actuator design

**Figure 2:**
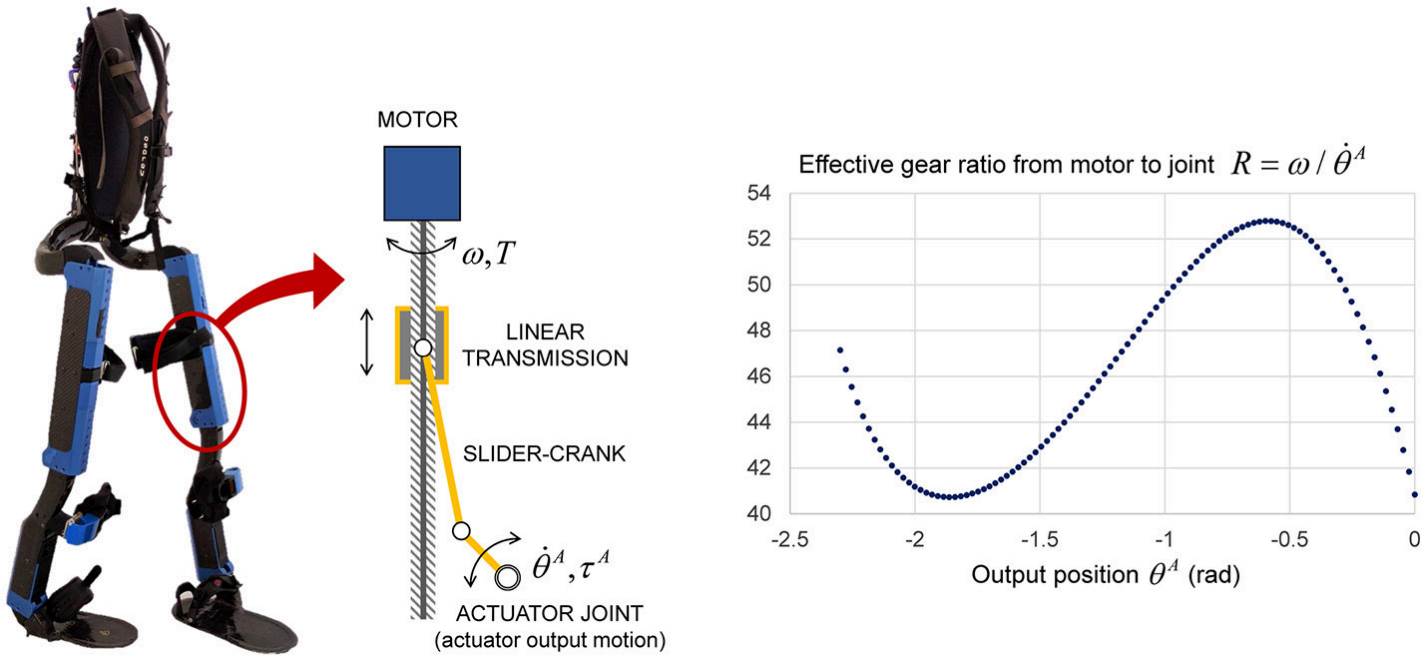
Schematic of the LLA used for all powered joints of Mina v2 (left). Effective gear ratio from motor rotation to joint rotation of each LLA (right).

The actuation of Mina v2 is modular in design. Each joint is powered by a custom Linear Linkage Actuator (LLA), allowing for ease of replacement, accessibility, and repair. The LLA was designed specifically for use with Mina v2, and features a frameless electric motor, integrated electronics, a load sensor, and an onboard motor amplifier and controller for distributed joint-level control. The motor, via a linear ball screw transmission, drives a slider-crank linkage mechanism connected to the joint output (Figure 2). The frameless motor has no internal gearing, i.e., its rotor is mounted on the same shaft as the ball screw, hence the effective gear ratio from the motor shaft to actuator joint output is $R=\omega /{\overset{\cdot }{\theta }}^{A}$, where ω is the rotational speed of the motor shaft and ${\overset{\cdot }{\theta }}^{A}$ is the actuator joint output velocity. Mechanical power losses in the linear transmission are negligible, given that the majority of the loss from the motor to the joint output occurs at the ball screw, which is typically 98–99% efficient. As a result, the effective joint output torque achievable by the actuator is estimated as τ^*A*^ = *TR*, where *T* is the motor torque.

The LLA exhibits a non-linear relationship between the motor position and the joint output position, resulting in an effective gear ratio *R* that varies with the stroke as a function of the output position θ^*A*^ (Figure 2). The values of the LLA's effective gear ratio *R* are calculated from the geometry and then verified experimentally by varying the output position within its admissible mechanical range in actuator space θ^*A*^ ∈ [−2.3, 0] (rad), where −1.25 rad corresponds approximately to mid-stroke configuration. As a result, the gear ratio varies between 41 and 53 as a function of the actuator output position, and is ~46 around mid-stroke.

Based on the 48 VDC bus voltage, the motor can achieve a maximum no-load speed of 3,340 rpm (ω_max_ = 349.76 rad/s) and a maximum stall torque *T*_max_ = 2.7 Nm due to thermal limitations. The motor limits and the effective gear ratio are used to obtain the joint output torque and velocity limits in actuator space as functions of joint position:

(1) $$\begin{array}{r} -\omega_{\max}/R\left(\theta^{A}\right)\leq {\overset{\cdot }{\theta }}^{A}\leq \omega_{\max}/R\left(\theta^{A}\right) \end{array}$$

(2) $$\begin{array}{r} -T_{\max}R\left(\theta^{A}\right)\leq \tau^{A}\leq T_{\max}R\left(\theta^{A}\right) \end{array}$$

**Figure 3:**
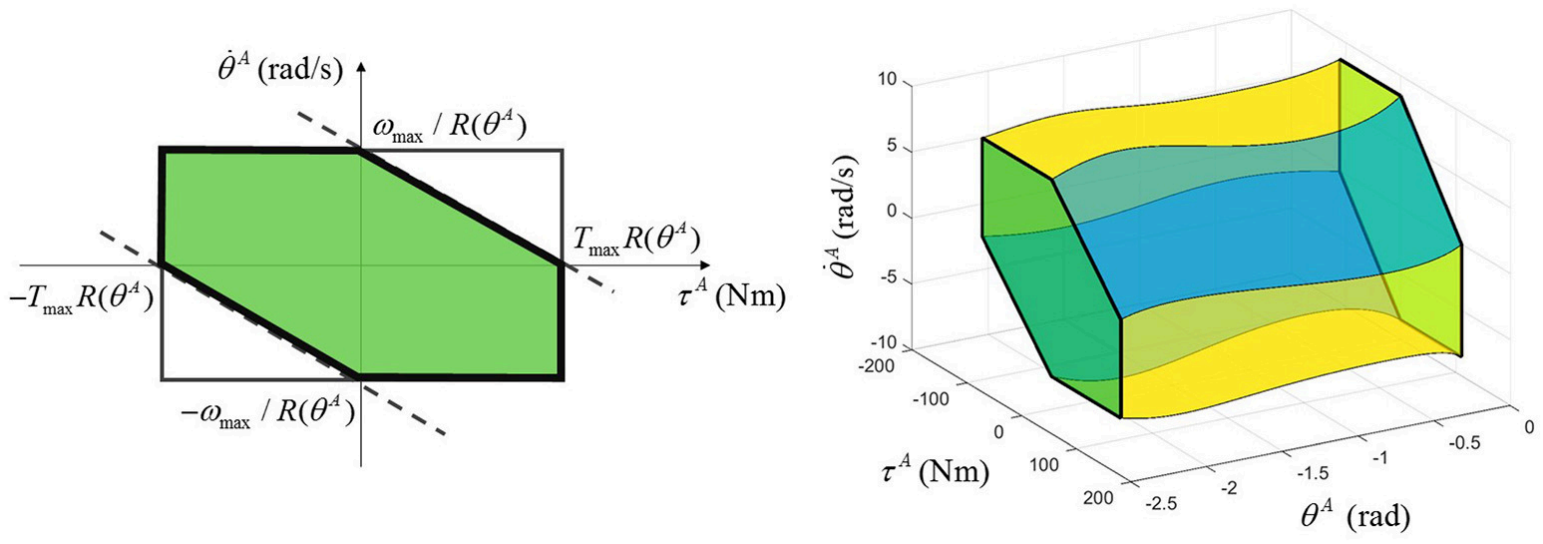
Multi-quadrant LLA torque-velocity limits for a given output position (left) and for the entire output position range (right).

For a given actuator joint output position θ^*A*^, the above lower and upper bounds define a rectangular region of actuator joint output torque-velocity ($\tau^{A},{\overset{\cdot }{\theta }}^{A}$) limits (Figure 3). In the first and third quadrants of this region, i.e., when the actuator performs positive work, the maximum and minimum velocities are also dependent on the torque, and are estimated as linear functions with intercepts at the peak no-load speed $\omega_{\max}/R\left(\theta^{A}\right)$ and peak torque $T_{\max}R\left(\theta^{A}\right)$ at the given joint output position. Hence, the feasible region of actuator joint output torque and velocity is additionally constrained by the following inequality:

(3) $$\begin{array}{r} -\omega_{\max}\leq {\overset{\cdot }{\theta }}^{A}R\left(\theta^{A}\right)+\frac{\omega_{\max}}{T_{\max}R\left(\theta^{A}\right)}\tau^{A}\leq \omega_{\max} \end{array}$$

When the actuator performs negative work (second and fourth quadrants), the speed of the motor is limited by the bus voltage, and the torque is limited by the rated current of the motor, and it is assumed that there is no additional relation between the speed and torque. Therefore, the four-quadrant torque-velocity feasible region in the actuator space takes the shape of a hexagon for a given output position and of a hexagonal-base volume for the entire range of joint output position (Figure 3).

### Exoskeleton mechanical model

**Figure 4:**
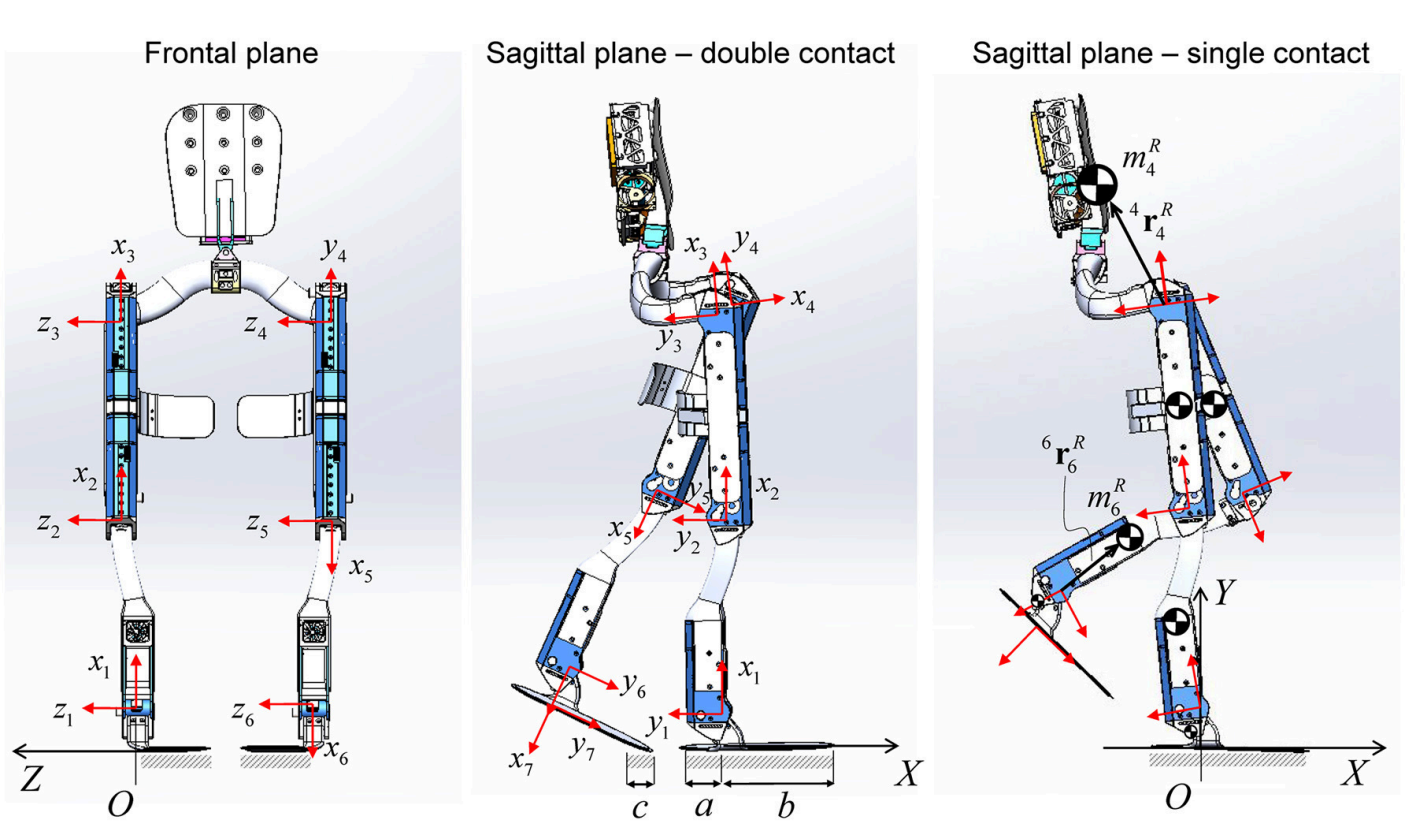
Mina v2 robotic exoskeleton design in the frontal and sagittal plane views. Local frame {*x*_*i*_, *y*_*i*_} for *i* = 1–7 is attached to each link, while the global frame {*X, Y, Z*} has origin *O* belonging to the region of the ground that is in contact with the stance foot. The COM of each link is shown. The orientation of the backpack is assumed to be always perpendicular to the *x*_4_ axis.

The mechanical design of Mina v2 is illustrated in the frontal and sagittal planes (Figure 4). Since this study focuses on the sagittal plane mobility and balance stability, the planar model of Mina v2 is described by a seven-link kinematic chain in the (*X, Y*) plane, with the origin at the center of the leading stance foot. The exoskeleton's mechanical design includes lower body links (feet, shanks, and thighs) and actuators, a pelvic belt, and a backpack containing a lithium ion battery (2.3 kg), computer, power distribution system, and networking hardware. The total mass of the backpack including the battery is 11.2 kg. From its mechanical design, the total mass of the exoskeleton is ~32 kg, while its actual mass including fasteners, wires, and pads (not included in the current model) may be slightly higher.

The local position of each robot link's COM (with mass $m_{i}^{R}$) is indicated by the position vector ^*i*^${\text{r}}_{i}^{R}$, relative to the local frame {*x*_*i*_, *y*_*i*_} attached to each link *i*, for *i* = 1–7 (Figure 4). In the sagittal plane model, a pelvic link with negligible length connects the hip joints (Mummolo et al., 2013) and has a total mass $m_{4}^{R}$ equal to the sum of the pelvic belt and the backpack masses, combined into one point mass located at ^4^${\text{r}}_{4}^{R}$.

The first and last links of the robot connect each ankle joint to its respective foot plate, and their length corresponds to the operator's foot height. The length of the foot plate is 33.4 cm, which is approximately equal to the operator's foot length, including the shoe. The lengths *a* = 0.099 m and *b* = 0.235 m are the distances in the sagittal plane from the projection of the ankle joint onto the ground to the rear and front edges of the foot plate, respectively. When the system is in single foot contact, the contact surface length is *a*+*b*, while, during double contact, it may vary; the foot plate is not rigid and has stiffness properties similar to those of a shoe. In the general double contact configuration, the dimension *c* of the contact patch at the trailing stance foot depends on the operator/controller strategy to move forward during the transfer phase of walking, as described later.

**Figure 5:**
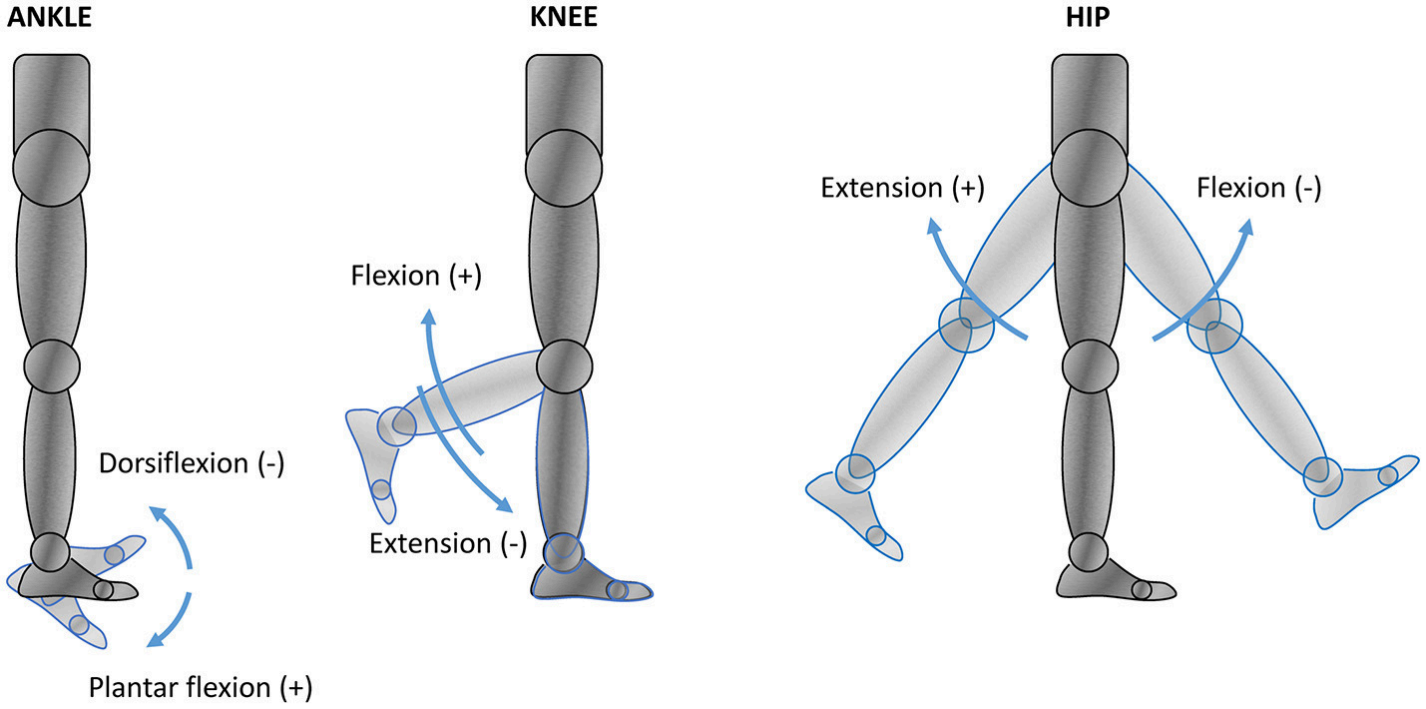
Lower body anatomical reference in joint space. The arrows indicate the positive and negative senses of rotation assumed by the current joint angle anatomical convention.

Due to different orientations of the LLAs within the exoskeleton structure and the joint angle conventions used (Figure 5), the LLA output must be mapped from the actuator space into the anatomical joint space of the robotic device, as follows:

(4) $$\begin{array}{r} \theta_{hip}^{R}=-\left(\theta^{A}+1.28\right);\theta_{knee}^{R}=-\theta^{A};\theta_{ankle}^{R}=\theta^{A}+1.05 \end{array}$$

(5) $$\begin{array}{r} \tau_{hip}^{R}=-\tau^{A};\tau_{knee}^{R}=-\tau^{A};\tau_{ankle}^{R}=\tau^{A} \end{array}$$

where superscripts *A* and *R* are used to indicate joint angles and torques in the actuator space and the robot (anatomical) joint space, respectively. Following the current lower body joint angle anatomical convention, positive angles θ^*R*^, velocities ${\overset{\cdot }{\theta }}^{R}$, and torques τ^*R*^ at the robot joints are used for hip extension, knee flexion, and ankle plantar flexion (Figure 5). The zero joint angles are the zero anatomical angles, which correspond to the angles observed in the upright standing pose on flat ground. In addition to the admissible mechanical range of the LLA output angle (θ^*A*^ ∈ [−2.3, 0] rad), the joint angles of Mina v2 are further constrained by more conservative limits, which are based on the operator's joint limits, to protect the operator from any extreme joint flexion and extension.

## Combined human-exoskeleton system: models and parameters

A seven-degree-of-freedom (DOF) model representing the combined human-exoskeleton system is established in the sagittal plane. The equivalent link, joint, and actuation parameters are derived by combining the planar models of the robotic exoskeleton and its human operator, both established in joint space.

### Models of the exoskeleton's operator

A seven-link model analogous to that used for the exoskeleton system describes the lower and upper body segments of Mina v2's human operator in the (*X, Y*) sagittal plane. The foot, shank, and thigh segments are modeled with three links for each leg. Lower body link lengths are directly measured from the human operator and used as a reference for modeling the exoskeleton's links such that Mina v2 and its pilot have identical link and foot lengths. The mass distribution of the human body is based on reference data from a biostereometric survey of six male subjects (Herron et al., 1976). The masses of human pelvis, torso, arm, and head segments are combined into one point mass located perpendicular to the pelvic link. Similarly to the exoskeleton model, the COM position of each link (with mass $m_{i}^{H}$) is described with respect to the local frame {*x*_*i*_, *y*_*i*_} by the position vector ^*i*^${\text{r}}_{i}^{H}$, for *i* = 1–7.

In this study, the operator has no volitional motor control of the lower limbs and the passive ranges of motion were measured by moving the joints gently until the ligaments provided resistance. Note that this same procedure can be done on subjects capable of voluntary motion, whose passive (or externally driven) ranges of motion will usually be larger than their active (internally driven) ones. The resulting joint ranges of motion are used as references for the design of safe mechanical limits for the robot.

Depending on the type and level of impairment, an appropriate model for internal joint torque at the human lower limbs should be formulated and combined with the robot's actuation model. The exoskeleton pilot is paraplegic and is assumed to exert no active torque at the lower body joints. In addition, internal torques caused by neuromuscular reflexes are not considered in this model, since the pilot's experience operating the robot suggests that such reflexes at the lower limbs tend to disappear over time with acclimation to the device. Therefore, the only joint torques at the human lower body segments are, in this case, due to the passive contribution of elastic elements. Each internal torque at the anatomical ankle, knee, and hip joints is modeled as a non-linear function of joint angle (Anderson et al., 2007):

(6) $$\begin{array}{r} \tau^{H}=B_{1}e^{k_{1}\theta^{H}}+B_{2}e^{k_{2}\theta^{H}} \end{array}$$

where the sign of θ^*H*^ follows the same anatomical reference used for the robot joint space (Figure 5). The parameters *B*_1_, *B*_2_, *k*_1_, and *k*_2_ for ankle, knee, and hip joints (Table 1) are obtained from a literature study (Anderson et al., 2007).

**Table 1:** Non-linear spring parameters for human passive joint torque models^a^.

**Human joint θ^**H**^**	***B*_1_**	***k*_1_**	***B*_2_**	***k*_2_**
Ankle	−0.0005781	5.819	0.967	−6.090
Knee	0	0	6.250	−4.521
Hip	−1.210	6.351	0.476	−5.910

a *The parameters shown represent the normative passive torque characteristics of healthy male subjects aged 18–25. More detailed subject- and impairment-specific characteristics of passive elements could be implemented in a similar manner, if such additional physiological data becomes available*.

### Equivalent DH model for the combined human-exoskeleton system

**Figure 6:**
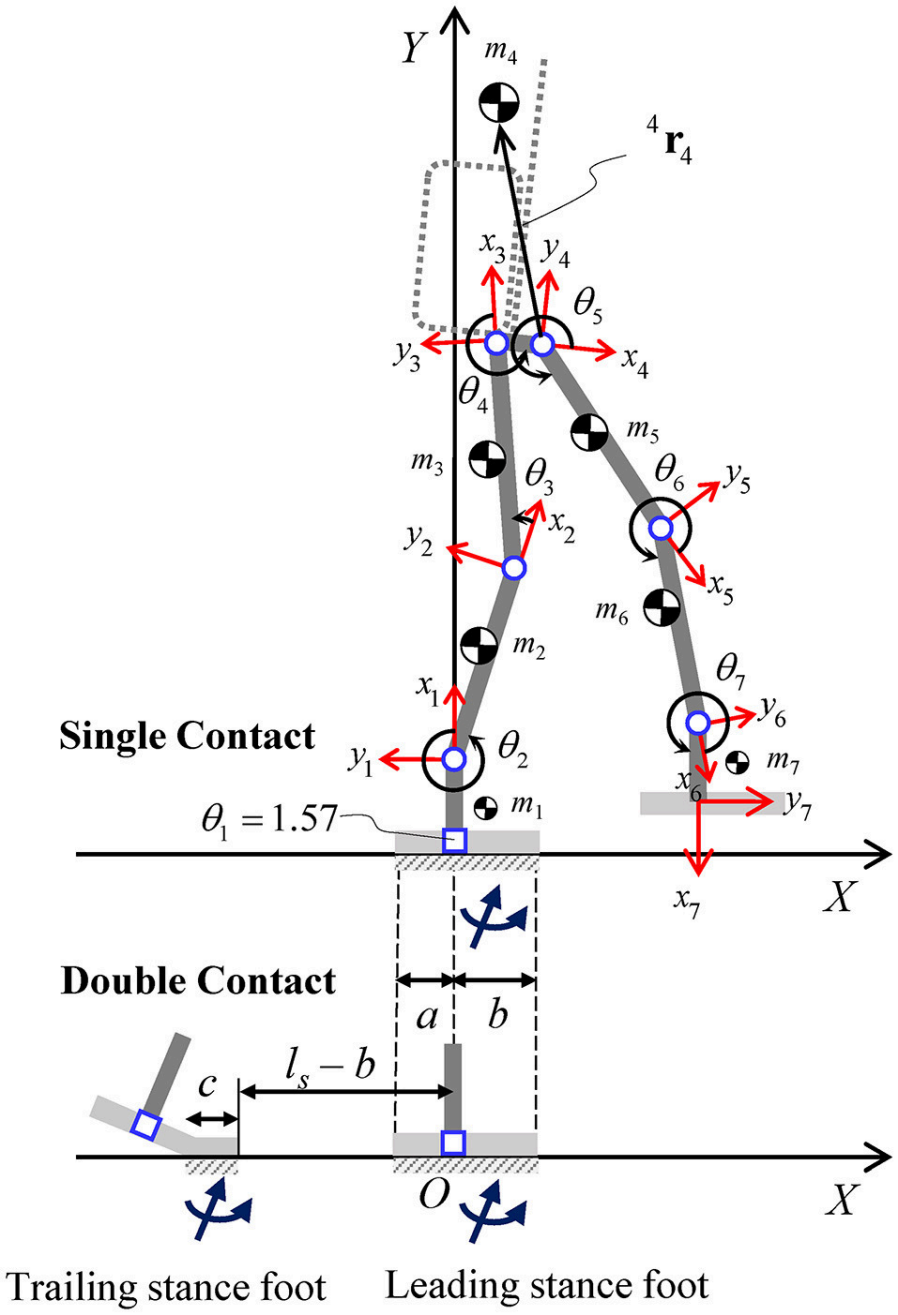
Planar model for the combined human-exoskeleton system in the sagittal plane. The links (thick solid lines) indicate the equivalent lower body segments (feet, shanks, and thighs) and the pelvis segment connecting the two hips. The local position of the equivalent COM of each link is described relative to the corresponding local frame {*x*_*i*_, *y*_*i*_}, for *i* = 1–7. The orientation of the backpack and upper body (dashed lines) is assumed to be perpendicular to link 4. Foot segments in single and double contact configurations are shown for reference, along with the corresponding contact area dimensions and resultant contact wrenches. In the double contact configuration, the distance between the front edges of the trailing and leading stance feet is equal to the step length (*l*_*s*_).

Based on the above-mentioned planar models for the robotic exoskeleton and the human body, an equivalent model is developed to represent the kinematics and dynamics of the combined human-exoskeleton system. The equivalent model in the sagittal plane consists of a 7-DOF serial kinematic chain, and thus can be established according to the Denavit-Hartenberg (DH) convention (Figure 6). Joints 2–7 are the revolute joints of the lower body, while joint 1, which connects the leading stance foot to the global frame {*X, Y*} origin, is fixed and has zero range of motion.

In this study, the seven corresponding exoskeleton and human links are combined into seven equivalent rigid bodies, assuming that the relative motion between the two systems is negligible and, therefore, θ^*H*^ = θ^*R*^ for each lower body joint. Since the exoskeleton's links and joint ranges of motion are designed based on the operator's body and joint parameters, the equivalent DH model has the same link lengths and joint limits as those of the robot. The equivalent link mass *m*_*i*_ of the combined system (Figure 6) is the sum of the *i*th link masses of the robot and human models, for *i* = 1–7. Point mass assumption is used to model the equivalent inertial parameters (COM location and inertia matrix) of each link, expressed with respect to the local frame {*x*_*i*_, *y*_*i*_} attached to link *i*. In particular, the local position of the equivalent point mass *m*_*i*_ relative to frame *i* is ^*i*^${\text{r}}_{i}=\left(m_{i}^{R}{\;}^{i}{\text{r}}_{i}^{R}+m_{i}^{H}{\;}^{i}{\text{r}}_{i}^{H}\right)/m_{i}$, from which the corresponding moments and products of inertia relative to frame *i* can be calculated.

The ankle, knee, and hip rotations of the equivalent model are described in joint space by the DH revolute joint variable θ_*i*_, which is measured from positive *x*_*i*−1_ to positive *x*_*i*_, counterclockwise by convention. The relationships between the DH joint variable θ_*i*_, for *i* = 2–7, and the lower body joint angles in the anatomical reference are given by:

(7) $$\begin{array}{l} \theta_{ankle}^{R}=\theta_{ankle}^{H}=\theta_{2}=-\theta_{7};\theta_{knee}^{R}=\theta_{knee}^{H}=\theta_{3}=-\theta_{6};\\ \theta_{hip}^{R}=\theta_{hip}^{H}=\theta_{4}+\pi /2=-\theta_{5}-\pi /2 \end{array}$$

Based on this transformation, the joint angle limits of the combined system, which are designed in the joint-space anatomical reference, can be expressed in the local reference of the equivalent model according to the DH representation.

In addition to the joint angle limits, the joint torque-velocity limits for the combined human-exoskeleton system must also be expressed in the joint space with respect to the DH joint variable θ_*i*_ and torque τ_*i*_, by taking into account the actuation limits of the robotic device (defined in the actuator space) and the passive joint torques in the human body (defined in the anatomical joint space). Using Equations (4) and (7), the joint variable θ_*i*_ is mapped into the actuator space through the relationships $\theta^{A}=f_{i}\left(\theta_{i}\right)$, for revolute joints 2–7, where:

(8) $$\begin{array}{l} f_{2}\left(\theta_{2}\right)=\theta_{2}-1.05\\ f_{3}\left(\theta_{3}\right)=-\theta_{3}\\ f_{4}\left(\theta_{4}\right)=-\left(\theta_{4}+\pi /2\right)-1.28\\ f_{5}\left(\theta_{5}\right)=\theta_{5}+\pi /2-1.28\\ f_{6}\left(\theta_{6}\right)=\theta_{6}\\ f_{7}\left(\theta_{7}\right)=-\theta_{7}-1.05 \end{array}$$

This mapping is used to model the robotic actuator's effective gear ratio in DH joint space, as a third-order polynomial function $R\left(f_{i}\left(\theta_{i}\right)\right)=c_{0}+c_{1}f_{i}\left(\theta_{i}\right)+c_{2}f_{i}{\left(\theta_{i}\right)}^{2}+c_{3}f_{i}{\left(\theta_{i}\right)}^{3}$, whose coefficients *c*_0_ = 41.14, *c*_1_ = −42.75, *c*_2_ = −47.03, and *c*_3_ = −12.84 are determined through curve fitting using the available *R* data (Figure 2). The joint velocity limits of the equivalent model as functions of the DH joint variable θ_*i*_ and its time derivative are formulated as follows:

(9) $$\begin{array}{l} -\omega_{\max}/R\left(f_{i}\left(\theta_{i}\right)\right)\leq \frac{df_{i}\left(\theta_{i}\right)}{dt}\leq \omega_{\max}/R\left(f_{i}\left(\theta_{i}\right)\right)\text{ for }i=\text{2}-\text{7} \end{array}$$

At a given joint, the sum of the robotic torque τ^*R*^ and the passive human torque τ^*H*^ in the joint-space anatomical reference provides the total actuation of the combined system, which can be mapped into the DH joint torque τ_*i*_ using the following relationships:

(10) $$\begin{array}{l} \tau_{ankle}^{R}+\tau_{ankle}^{H}=\tau_{2}=-\tau_{7};\tau_{knee}^{R}+\tau_{knee}^{H}=\tau_{3}=-\tau_{6};\\ \tau_{hip}^{R}+\tau_{hip}^{H}=\tau_{4}=-\tau_{5} \end{array}$$

where positive torques τ_*i*_ in the DH local reference frames follow the right hand rule. Using Equations (5) and (10), the robotic torques in actuator space can be expressed as functions of the DH joint variables and torques through the mapping $\tau^{A}=\phi_{i}\left(\tau_{i},\theta_{i}\right)$, for *i* = 2–7, as follows:

(11) $$\begin{array}{l} \phi_{2}\left(\tau_{2},\theta_{2}\right)=\tau_{2}-\tau_{ankle}^{H}\left(\theta_{2}\right)\\ \phi_{3}\left(\tau_{3},\theta_{3}\right)=-\left(\tau_{3}-\tau_{knee}^{H}\left(\theta_{3}\right)\right)\\ \phi_{4}\left(\tau_{4},\theta_{4}\right)=-\left(\tau_{4}-\tau_{hip}^{H}\left(\theta_{4}+\pi /2\right)\right)\\ \phi_{5}\left(\tau_{5},\theta_{5}\right)=\tau_{5}+\tau_{hip}^{H}\left(-\theta_{5}-\pi /2\right)\\ \phi_{6}\left(\tau_{6},\theta_{6}\right)=\tau_{6}+\tau_{knee}^{H}\left(-\theta_{6}\right)\\ \phi_{7}\left(\tau_{7},\theta_{7}\right)=-\tau_{7}-\tau_{ankle}^{H}\left(-\theta_{7}\right) \end{array}$$

where the human passive torques are written as functions of θ_*i*_ using the transformations in Equation (7).

Based on the mappings *f*_*i*_(θ_*i*_) and ϕ_*i*_(τ_*i*_, θ_*i*_), the LLA output torque limits can be rewritten as functions of the DH joint and torque variables θ_*i*_ and τ_*i*_, for *i* = 2–7:

(12) $$\begin{array}{l} -T_{\max}R\left(f_{i}\left(\theta_{i}\right)\right)\leq \phi_{i}\left(\tau_{i},\theta_{i}\right)\leq T_{\max}R\left(f_{i}\left(\theta_{i}\right)\right) \end{array}$$

Lastly, the linear relationship in the first and third quadrants between actuator output velocity and torque at each joint can be rewritten as a function of DH joint variable (and its derivative) and torque, as follows:

(13) $$\begin{array}{l} -\omega_{\max}\leq \frac{df_{i}\left(\theta_{i}\right)}{dt}R\left(f_{i}\left(\theta_{i}\right)\right)+\frac{\omega_{\max}}{T_{\max}R\left(f_{i}\left(\theta_{i}\right)\right)}\phi_{i}\left(\tau_{i},\theta_{i}\right)\leq \omega_{\max} \end{array}$$

The equivalent DH model, along with the above link inertial parameters, joint transformations, and actuation model, is used to formulate the kinematics, dynamics, and the corresponding constraints of the combined human-exoskeleton system. In this study, the recursive Lagrangian dynamics is used to derive the joint-space equations of motion of the equivalent DH model.

## System control and experiments

The exoskeleton-assisted gait is generated using pre-defined reference joint angle trajectories for the hips, knees, and ankles, based on the upcoming footstep locations and the type of terrain to be traversed (flat ground, steps, or slopes). Within one step of the walking cycle, the exoskeleton's contact configurations with the ground are double contact (during the transfer phase) and single contact (during the swing phase), while the operator is always allowed to make additional contacts with the ground by placing the crutches.

**Figure 7:**
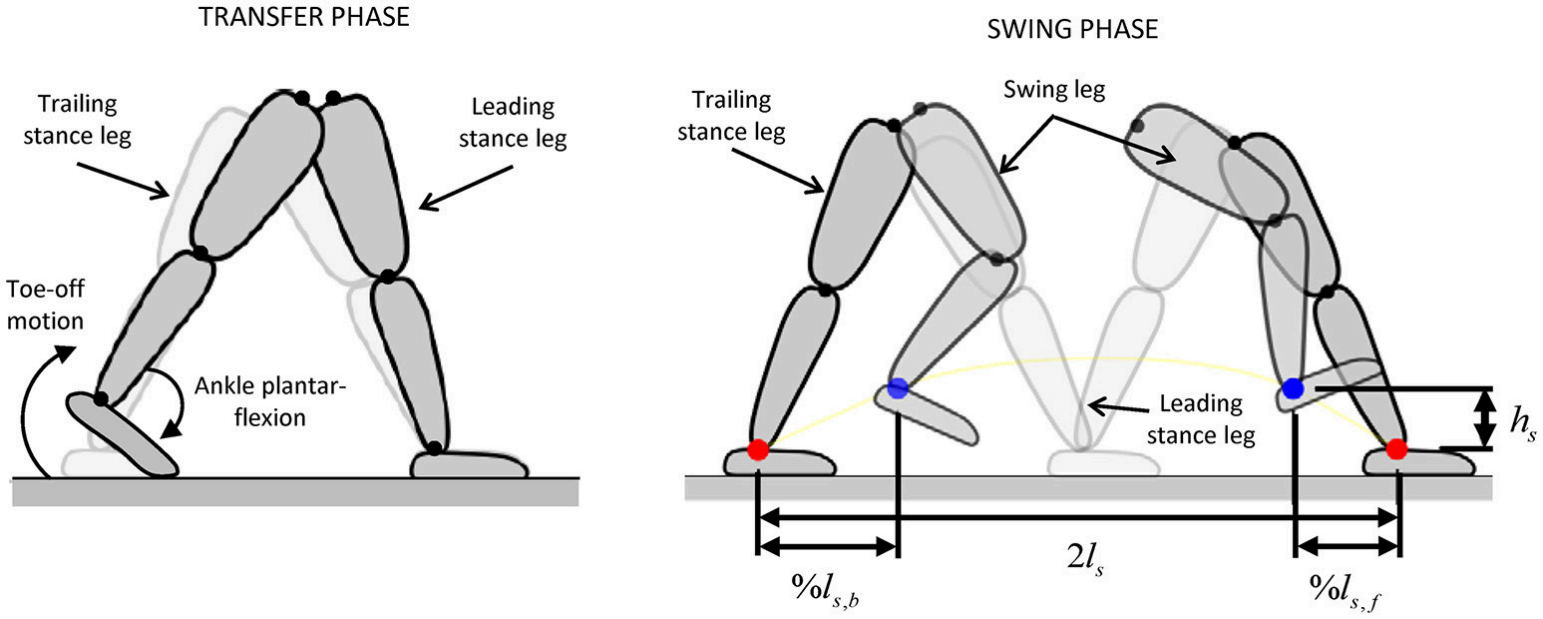
Joint trajectory planning for transfer and swing phases [adapted from Griffin et al. (2017)]. Tuning parameters %*l*_*s, f*_, *%l*_*s, b*_, *l*_*s*_, and *h*_*s*_ are selected to generate the desired joint motions in the swing phase.

During the transfer phase of walking (Figure 7), a toe-off movement is designed in order to exploit the presence of the powered ankle joints in Mina v2 (Griffin et al., 2017). In particular, a minimum jerk trajectory is planned for the trailing ankle joint, such that it reaches a given final plantar flexion angle at the end of transfer, while the body and the leading leg rotates about the leading ankle. This ankle plantar flexion during toe-off motion is powered by the ankle actuator and provides a forward force to the body and helps the push-off of the trailing stance foot prior to initiation of swing. As a result of the reference trajectories for continuous walking, the trailing stance foot during the transfer phase is mostly in toe contact, resulting in a contact region with an approximate dimension *c* = 8 cm due to the flexibility of the shoe and the foot plate (Figure 6).

During the swing phase, four Cartesian-space waypoints are defined for the swing foot: the starting position, the upcoming foothold at a distance equal to the stride length (2*l*_*s*_), and two midpoints positioned at fractions %*l*_*s, b*_ and *%l*_*s, f*_ of the nominal step length (*l*_*s*_) and at a fixed height (*h*_*s*_) (Figure 7). The parameters %*l*_*s, b*_, *%l*_*s, f*_, *l*_*s*_, and *h*_*s*_ are tuned for a given step, while the joint angles at each waypoint are calculated using inverse kinematics. The reference joint trajectories are formulated as the minimum jerk trajectories passing through each of these waypoints. The combination of the flexible trajectory design during swing phase with the use of powered toe-off motion contributed significantly to the system's successful gait performance (Griffin et al., 2017).

For the exoskeleton to execute the generated walking trajectory, each actuator is operated in position control mode on the Elmo Twitter Gold embedded motor controller. This motor amplifier closes the position loop using current control to account for position error at a loop rate of ~3 kHz. The resulting joint-level behavior produces the highest-achievable impedance actuation at each joint, tracking positions as best as possible. Using position control as the basis for the motion comes at the cost of low compliance between the device and the terrain.

**Figure 8:**
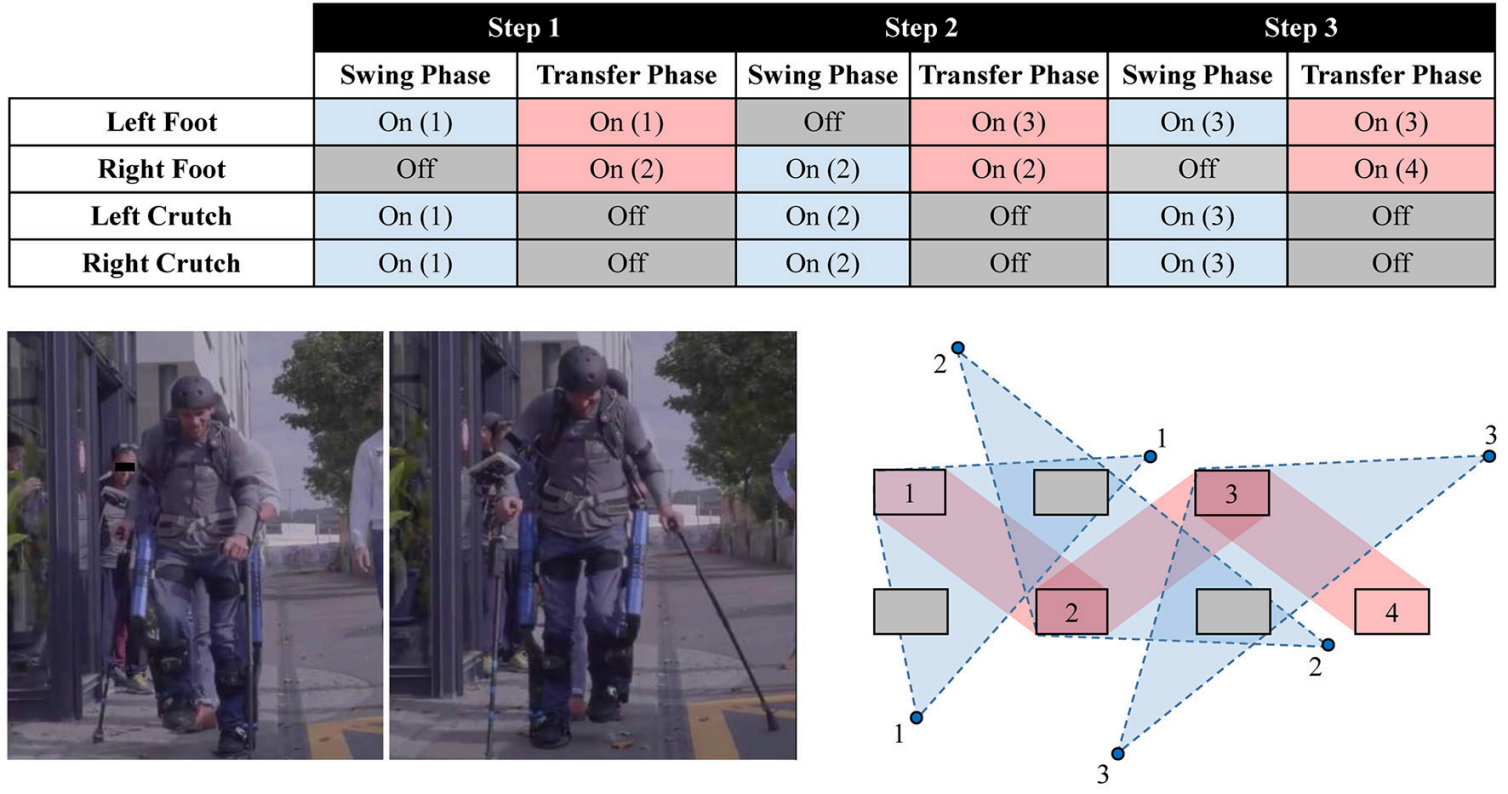
The contact sequence used by the operator in the gait phases. The numbered rectangles and circles indicate the locations of feet and crutches placement, respectively. The support polygon during the swing and transfer phases is shown by the regions with dashed and solid border, respectively. The contact sequence shown corresponds to three complete steps (permission for image reproduction granted by the participant).

The operator was not given specific performance instructions other than to execute a typical walking gait. While walking, the crutches are repositioned during every transfer phase, and are placed on the ground during the swing phase. The operator adopts a tripod-type gait during swing phase, balancing on one leg and two crutches, and always moves both crutches during the transfer phase, standing stably on both legs (Figure 8). The phase changes of the controller were entirely governed by user-selected time, and did not, nor could, rely on any contact or force sensor.

The joint trajectories of the combined human-exoskeleton system were recorded by the exoskeleton's motor encoders for over 20 trials of flat-ground continuous walking experiments. The data was averaged over all the walking steps taken during the trials, representing 80 steps. The average robot joint kinematics (angular positions ${\theta_{i}}^{R}\left(t\right)$ and velocities ${{\overset{\cdot }{\theta }}_{i}}^{R}\left(t\right)$) measured across the walking trials is mapped into the DH kinematics ($\theta_{i}\left(t\right),{\overset{\cdot }{\theta }}_{i}\left(t\right)$) and used to evaluate the forward COM kinematics of the equivalent DH model. In particular, the sagittal plane global position $\bar{r}\left(t\right)$ and velocity $\overset{\cdot }{\bar{r}}\left(t\right)$ of the combined system's COM are calculated at all times as functions of θ_*i*_(*t*) and ${\overset{\cdot }{\theta }}_{i}\left(t\right)$, where the kinematic chain's global frame has its origin at the center of the leading stance foot, which is constrained to be flat on the ground during the entire step duration.

## Contact-dependent balance stability analysis

The state-based stability is evaluated for Mina v2's robot-assisted balance and locomotion. A numerical optimization framework is used to construct the balance stability boundaries (BSBs) of the combined human-exoskeleton system in single and double legged supports.

### Dynamic model with contact constraints

The BSBs of the combined human-exoskeleton system are constructed by iteratively solving a series of constrained non-linear optimization problems, in which the joint-space constrained dynamics of the equivalent DH model is implemented. The joint-space equations of motion are recursively formulated for the equivalent DH model in its open- (single contact) and closed-loop (double contact) kinematic configurations, by taking into account the dynamics of the contact interactions between the system and its environment. The contact dynamics of the single and double contact configurations are the results of the kinematic and kinetic constraints at the feet imposed during the swing and transfer phase of walking, respectively.

Within one complete step cycle, the center of the leading stance foot is fixed at the origin of the global frame {*X, Y*} and its orientation is coincident with that of the ground plane at all instants in time. For both contact configurations, no relative motion between the contact surface of the stance feet and the ground is allowed. During the transfer phase, the front edge of the trailing stance foot is fixed to a point with *X*-coordinate equal to –(*l*_*s*_ – *b*), consistent with the step length (Figure 6). The orientation of the trailing stance foot about the metatarsal joint [positioned at *X*-coordinate of –(*l*_*s*_ – *b* + *c*)] is left free, as it rotates during the toe-off motion. During the swing phase, the stance foot remains in full contact with the ground, while the *Y*-coordinate of the swing foot and any other part of the system is constrained to be above the ground level.

The distributed reaction forces at the contact interface between the feet and the ground are modeled with one equivalent system of resultant contact force and moment (i.e., contact wrench) applied at each stance foot (Figure 6). The resultant contact wrench is null at the swing foot during the single contact configuration (e.g., swing phase), while the contact wrench at the support foot is uniquely determined for a given motion. Therefore, in single contact, the unknowns of the non-linear optimization problems for BSB construction are joint trajectories, while joint torques and the reactions at the fixed base are recursively determined from the inherent inverse dynamics scheme. In the double contact configuration (e.g., transfer phase), the distribution of contact wrenches between the two feet is indeterminate, and the contact wrenches, joint kinematics, and actuator torques must all be solved for within the given optimization problem. In this study, the unknowns of the optimization problems for the construction of the BSB in double contact are joint trajectories and contact wrenches at both feet, while joint torques are again determined from inverse dynamics. This formulation in double contact is based on the conjunction of joint- and COM-space dynamics of the given biped system [full details available in Mummolo et al. (2018)].

In addition, the kinetic constraints related to the ground reaction forces and moments applied at each foot are imposed. For each contact surface, the resultant contact force in the normal direction is subject to the unilateral constraint ensuring that the ground only exerts positive normal forces that push on the foot. The friction cone constraint is also imposed on the tangential component of the resultant reaction force to prevent sliding. The position of the center of pressure calculated for each contact wrench is constrained to be within the contact area dimension of the corresponding foot in the sagittal plane (i.e., *c* for the trailing stance foot and *a* + *b* for the leading stance foot) to ensure that any physically realizable pressure distribution (and the corresponding resultant contact wrench) does not cause the foot to tip over.

### Balance stability boundary construction

The balancing capability of a legged system is a characteristic that is dependent on the system's current state (position and velocity) and its current contact configuration (Mummolo et al., 2017). In this study, the capability of the combined human-exoskeleton system to maintain balance solely through the support of the legs (i.e., without crutches) is quantified in the sagittal plane, thus isolating the role of crutches in assisting stability in the fore-aft (+*X* and –*X*) directions. A state-based stability criterion for legged systems that was recently introduced (Mummolo et al., 2018) is used to evaluate the balancing capabilities of Mina v2 in its two main foot-ground (single and double) contact configurations. In the proposed criterion, the equivalent DH model's COM state as its global Cartesian position $\bar{r}\left(t_{0}\right)$ and velocity $\overset{\cdot }{\bar{r}}\left(t_{0}\right)$ at a given time *t*_0_ is used to determine whether the system is in a balanced state with respect to a specified contact configuration, according to the definitions in the authors' previous study (Mummolo et al., 2018). In other words, if the legged system can reach a static equilibrium from a current state $\left(\bar{r}\left(t_{0}\right),\overset{\cdot }{\bar{r}}\left(t_{0}\right)\right)$ without ever altering its contact configuration, that state is *balanced* with respect to that contact configuration. Vice versa, if the current state $\left(\bar{r}\left(t_{0}\right),\overset{\cdot }{\bar{r}}\left(t_{0}\right)\right)$ leads to an inevitable change in the system's current contact configuration, the state is *unbalanced* with respect to that contact configuration.

The implementation of the proposed COM state-based criterion consists in the numerical construction of the system-specific and contact-specific BSB. The BSB is a partition of the COM state space that includes all possible balanced states of the given system in the specified contact configuration. A COM state outside of the BSB represents the sufficient condition for losing balance, from which a change in the system's contact is inevitable. For each sampled COM initial position $\bar{r}\left(t_{0}\right)={\bar{r}}^{*}\left(t_{0}\right)$, the optimal trajectories and actuator torques in the joint space are found, such that the component of the initial COM velocity $\overset{\cdot }{\bar{r}}\left(t_{0}\right)$ along a desired direction is maximized while satisfying relevant constraints. From the joint-space solution of each optimization problem, the state $\left({\bar{r}}^{*}\left(t_{0}\right),{\overset{\cdot }{\bar{r}}}^{*}\left(t_{0}\right)\right)$ is calculated and stored as a point of the BSB, and represents the most extreme balanced state for the system at the given COM position and in the specified contact configuration.

At each iteration of this numerical construction algorithm, a new COM position is sampled within the system's contact-specific COM workspace, which is the region of all positions reachable by the legged system's COM subject to joint limits and the specified kinematic contact constraints. In this study, a rectangular grid with a uniform spacing is used to sample the entire workspace area, while any general discretization strategy can be used. In each optimization problem, the equivalent DH model is governed by the dynamics in joint and COM spaces, including the contact dynamics formulation, and is subject to the following constraints:

- *System-specific design*: combined joint torque-velocity limits [Equations (9, 12, 13)] and equivalent link and mass parameters.
- *Contact kinematics*: global position and orientation constraints for each foot segment in the single and double contact configurations.
- *Contact kinetics*: unilateral normal reaction force, friction cone, and center of pressure limits for each foot.
- *Balanced state conditions*: sampled initial COM position, preservation of the given contacts, and long-term (at a sufficient final time *t*_*f*_) static equilibrium of the COM.

The resulting initial conditions $\left({\bar{r}}^{*}\left(t_{0}\right),{\overset{\cdot }{\bar{r}}}^{*}\left(t_{0}\right)\right)$, for all possible sampled initial COM positions, are the points of the BSB in the state space and identify the maximum allowable COM velocity perturbations along +*X* and –*X* directions for the given system such that static equilibrium can still be reached while remaining solely in the specified (single or double) contact configuration.

## Results and discussion

The established parameters are integrated into the combined human-exoskeleton system model. The stability boundaries constructed for the combined system in legged supports are analyzed in the system's COM state space. Then the states of balance of the robot-assisted walking motion are evaluated against the BSBs.

### Combined system model and walking trajectories

Mina v2's design was customized for its pilot, and the joint positions and link lengths of the exoskeleton model closely match those of the human operator (Table 2). The human subject operating Mina v2 in the current experiments is a male, is 1.78 m tall, and has a total mass of 82.8 kg. This mass is similar to that of Subject 2 from a literature study (Herron et al., 1976), which is used to estimate the mass distribution of the operator's body segments. The link and mass parameters for the equivalent DH model of the combined human-exoskeleton system were calculated accordingly (Table 2). In particular, the equivalent link's COM local position ^*i*^**r**_*i*_ is calculated from ^*i*^${\text{r}}_{i}^{R}$ and ^*i*^${\text{r}}_{i}^{H}$, for *i* = 1–7, using the proposed methods as described above.

**Table 2:** Link parameters for the sagittal plane model of Mina v2, the operator, and the equivalent DH model for the combined human-exoskeleton system.

**Link number**	**Body part**	**Link length (m)**	**Link mass (kg)**			**Link's local COM position vector (*x_*i*_*, *y_*i*_*) (cm)**		
			**Robot**	**Human model**	**Equivalent DH model**	**Robot**	**Human model**	**Equivalent DH model**
			**$m_{i}^{R}$**	**$m_{i}^{H}$**	***m_*i*_***	**^*i*^${\text{r}}_{i}^{R}$**	**^*i*^${\text{r}}_{i}^{H}$**	**^*i*^**r**_*i*_**
1	Right foot height	0.087	0.760	1.209	1.969	(−5.89, −3.44)	(−6.55, −5.08)	(−6.30, −4.45)
2	Right shank	0.422	2.902	3.234	6.136	(−26.19, −2.94)	(−17.99, 0.00)	(−21.87, −1.39)
3	Right thigh	0.424	5.206	8.378	13.584	(−20.89, −3.36)	(−16.32, 0.00)	(−18.07, −1.29)
4	Upper body^*^	0.001	2.953 + 11.2	57.177	71.330	(−15.38, 17.25)	(−0.88, 37.63)	(−3.75, 33.59)
5	Left thigh	0.424	5.206	8.378	13.584	(−21.50, 3.36)	(−26.08, 0.00)	(−24.33, 1.29)
6	Left shank	0.422	2.902	3.234	6.136	(−16.01, 2.94)	(−24.21, 0.00)	(−20.33, 1.39)
7	Left foot height	0.087	0.760	1.209	1.969	(−2.81, 3.44)	(−2.15, 5.08)	(−2.40, 4.45)

* *The robot's upper body consists of a pelvis link (2.953 kg) and a backpack (11.2 kg). The human's upper body includes the head, arms, torso, and pelvis*.

The operator's (passive) joint ranges of motion were obtained as previously described and the robot joint limits are designed in the anatomical joint space to be less than or equal to the operator's joint range of motion, with a safety margin as a precaution. Based on these ranges of motion and the transformations in Equation (7), the lower and upper bounds of the joint variables in the equivalent DH model are expressed with respect to the local reference frames (Table 3).

Table 3Joint ranges of motion (in degrees) of Mina v2, the operator, and the combined human-exoskeleton system.

**Table:** 

**Anatomical reference**						
	**Human**		**Robot**		**Combined system**	
**Joint**	**Flexion**	**Extension**	**Flexion**	**Extension**	**Flexion**	**Extension**
Ankle	−34 (dorsiflex.)	51.5 (plantar flex.)	−30 (dorsiflex.)	40 (plantar flex.)	−30 (dorsiflex.)	40 (plantar flex.)
Knee	122	0	118	0	118	0
Hip	−140	45	−105	0	−105	0

**Table:** 

**Local DH reference for combined system**							
**Joint**		**Lower bound**	**Upper bound**	**Joint**		**Lower bound**	**Upper bound**
Ankle	θ_2_	−30	40	Ankle	θ_7_	−40	30
Knee	θ_3_	0	118	Knee	θ_6_	−118	0
Hip	θ_4_	−195	−90	Hip	θ_5_	−90	15

The resulting dynamic models of the combined human-exoskeleton system are implemented into the optimization problems for the BSB construction. As a simple measure of model validation, the total normal component of the ground reaction force(s) is equal to 1,124.9 N at the final static equilibrium for all single and double contact solutions to the optimization problems, which accurately reflects the weight of the combined system with a total mass of 114.7 kg.

**Figure 9:**
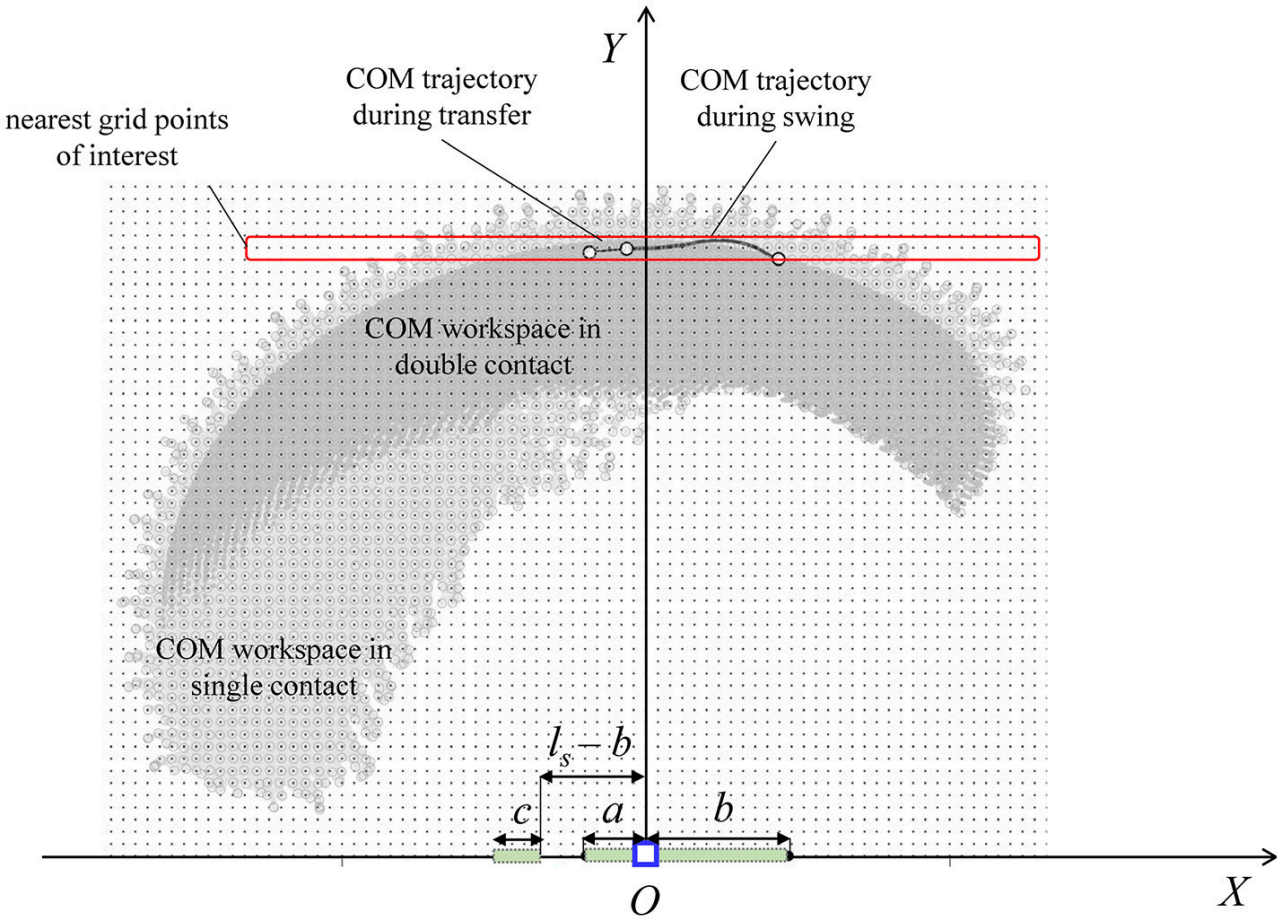
Contact-specific COM workspaces, discretized using a rectangular grid for COM initial position sampling in the construction of the BSB. The COM trajectory in the sagittal plane is calculated from the average joint angle experimental data of one step walking cycle. The contact dimensions during single (*a* + *b*) and double (*a* + *b* and *c*) contacts are shown.

A forward kinematics algorithm processes the link parameters (*m*_*i*_ and ^*i*^**r**_*i*_; Table 2), the DH joint angle limits (Table 3), and the kinematic contact constraints of the equivalent DH model, and evaluates the system's contact-specific COM workspace in the single and double contact configurations (Figure 9). The workspace area in single contact is larger than that in double contact. In particular, since the double contact configuration satisfies all kinematic constraints present in the single contact configuration plus the additional constraint of the trailing stance foot position, every joint configuration that satisfies the double contact kinematic constraints also satisfies those of the single contact configuration. As a result, the double contact workspace area is entirely included inside the single contact workspace area.

**Figure 10:**
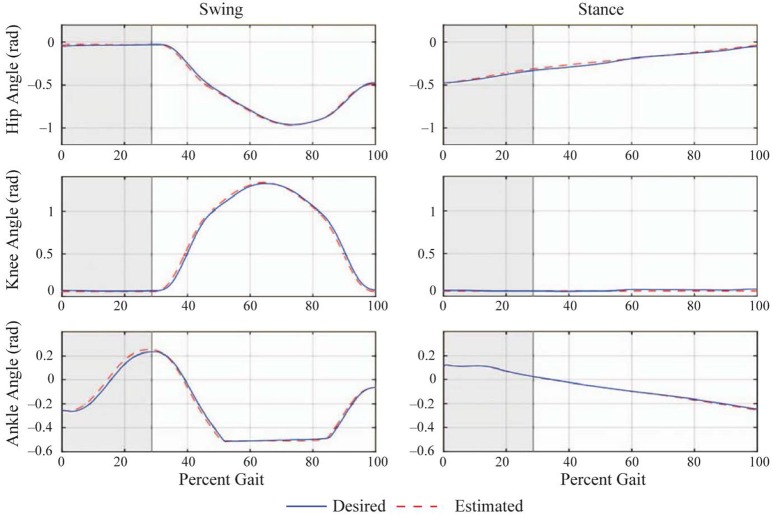
Average joint angle trajectories during one step of the flat ground walking trials [adapted from Griffin et al. (2017)]. The desired trajectories are shown as solid (blue) and the actual trajectories are shown as dashed (red) lines. The horizontal axis (percent gait) is the time axis normalized by the duration of a step cycle. The shaded region represents the double contact transfer phase. The plots in the left column are the joint angles of the trailing stance leg that performs the swing motion, and those in the right column are for the leading stance leg, which is always in contact with the ground during a step. When the percent gait reaches 100%, the legs switch roles, such that a new step cycle begins.

The experimental joint trajectories were averaged for one complete step (Figure 10) and correspond to the robot and human joint rotations in the sagittal plane. The nominal step length corresponding to the reference joint angle trajectories for each walking trial is *l*_*s*_ = 0.4 m, with %*l*_*s, b*_ = %*l*_*s, f*_ = 30, *h*_*s*_ = 0.1 m, and the swing and transfer time equal to 1.0 s and 0.4 s, respectively. The average walking speed of the trials was 0.29 m/s. The forward kinematics algorithm also processes the average joint trajectories [mapped into the DH kinematics ($\theta_{i}\left(t\right),{\overset{\cdot }{\theta }}_{i}\left(t\right)$)] for the calculation of the corresponding average COM trajectory of the combined human-exoskeleton system during the flat-ground walking experiments. The total COM trajectory in the sagittal plane during walking (plotted for one step in Figure 9) is included within and close to the workspace boundaries corresponding to the single and double contact configurations, with an average *Y*-coordinate of 1.00 m. In this study, the COM positions for the BSB construction were sampled within the workspace at the grid points nearest to the COM trajectory in the (*X, Y*) plane, in order to characterize the system's balance stability at a COM height similar to that of experimental walking trials. Therefore, the selected sample points for the COM initial position $\bar{r}(t_{0})={\bar{r}}^{*}(t_{0})$ have a *Y*-coordinate of 1.00 m and an *X*-coordinate within the corresponding workspace ranges of [−0.368, 0.4255] for single contact and [−0.162, 0.148] for double contact (in meters), with uniform spacing of 2 cm.

### Balance stability boundaries for legged support

**Figure 11:**
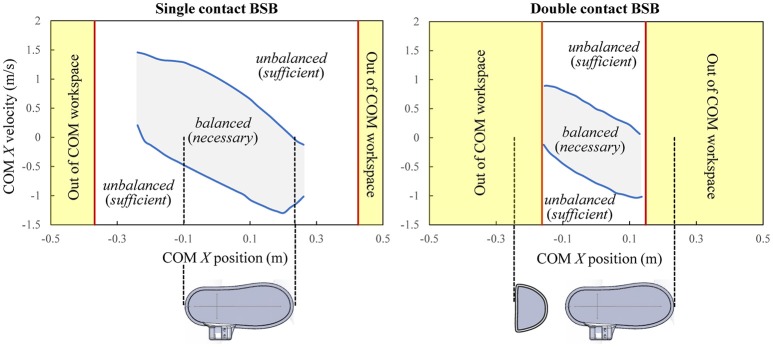
Balance stability boundaries during single and double contacts. Note that a COM state being inside or on the BSB is a necessary condition for the system to be currently balanced, while it being outside of the BSB is the sufficient condition for unbalanced.

The balance stability characteristics of the combined human-exoskeleton system in legged support are demonstrated through the calculation of the BSB for single and double contact configurations. The velocity extrema are found along the +*X* and –*X* directions to evaluate the stability characteristics of the combined system in the sagittal plane against positive and negative perturbations along the direction of forward walking progression. The BSB results corresponding to the selected grid points of interest are projected onto the X-state space (Figure 11).

The BSBs quantify of the state space regions within which the combined system can maintain balance using only the support of the leg(s) and without resorting to other balancing mechanisms, such as crutch placement. If the *X* component of any velocity perturbation of the combined system's COM are within the single contact BSB threshold (Figure 11, left), it indicates that balance can be maintained on a single foot and without crutches. If the *X* component of a velocity perturbation of the combined system's COM surpasses the single contact BSB threshold, then the human-exoskeleton system will not be able to stop unless the single contact is altered, for example, by placing the non-stance foot (i.e., stepping) or crutches on the ground. In this case, the current COM state is said to be an unbalanced state with respect to the specified single contact configuration, and will necessarily end up in a contact change. A similar statement can be made for the double contact BSB results (Figure 11, right). Note that the BSBs results are not associated to a specific motion, and their construction algorithm does not assume any specific controller design. Instead, the state space partitions identified by the BSBs are the result of the system properties (mechanical and actuation models) and the specified contacts with the environment.

The states of balance for each contact configuration can be analyzed with respect to the *X* limits of the COM workspace at *Y* = 1.00 m and the *X* dimensions of the base of support between the foot/feet and the ground (Figure 11). For the single contact, balanced states exist for the COM positions only within the range [−0.24, 0.26], in meters, which is smaller than its workspace range (Figure 11, left). While there are COM positions out of this range that are kinematically feasible within the single contact COM workspace, they cannot be balanced due to kinetic constraints. When the COM position lies sufficiently outside of the foot base of support, regardless of its velocity, restoring balance requires either the motion of the stance foot (sliding or tipping-over) relative to the ground or the presence of additional contacts (stepping or crutch placement); otherwise, falling is inevitable. For the double contact configuration, the BSB extends up to its workspace limits. Therefore, all COM positions within the double contact COM workspace can be balanced if their velocity perturbations in the fore-aft directions are within the double contact BSB. In addition, all COM positions within the double contact BSB are also statically stable, in other words, all their *X*-positions are within the base of support region. Note that a statically stable COM position can be inside or outside of the BSB (i.e., balanced or unbalanced) depending on its COM velocity.

The single contact BSB is much larger than and encloses the double contact BSB, and therefore has a greater balancing capability. In a single contact, the freedom of the system to employ its angular momentum enhances its balancing performance. For the double contact configuration, the balancing advantage of the additional contact wrench at the trailing stance foot is offset by the condition for double contact that both feet must be pinned to the ground. However, it should be noted that some unbalanced double contact states may result in being within the single contact BSB through a foot detachment, and thus can maintain balance without crutch placements.

### Balance stability of robot-assisted gait and role of crutches

**Figure 12:**
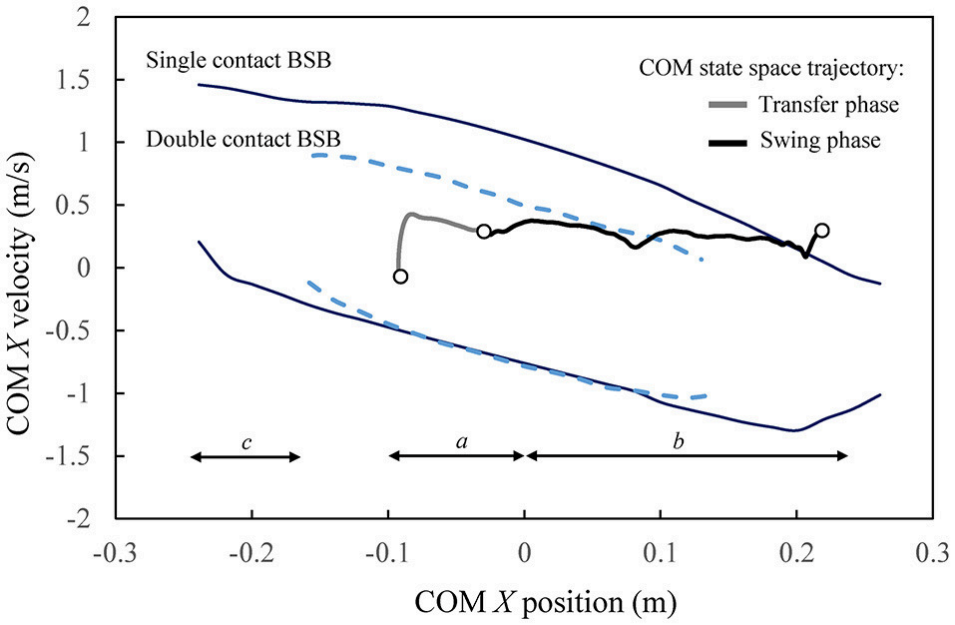
Crutch-less balance stability characteristics of the robot-assisted crutched walking trajectories. The forward walking progression is in the positive *X* direction. Markers indicate the beginning and the end of the transfer and swing phases.

The BSBs under the support of the leg(s) and in the absence of additional contacts, such as crutches, can serve as a basis for comparison when characterizing the balance stability of the current robot-assisted walking motion during transfer and swing phases. In particular, the balance stability of the average COM state space trajectory during one step obtained from the flat ground walking trials is analyzed with respect to the contact-specific BSB for the corresponding gait phases (Figure 12).

The COM state space trajectory from the experimental walking trials is the result of the gait planning and control implemented as described previously, which was formulated in the lower body joint space without any balance controller, and thus resulted in the use of crutches. It is observed that the COM state trajectory during transfer and swing mostly lies within the corresponding BSB (double and single contact, respectively). The transfer phase begins and ends well within the double contact BSB, indicating that each state of the prescribed transfer trajectory is balanced with respect to the double contact configuration. This implies that a control strategy could be designed such that the same transfer motion can be performed stably (at least with respect to the sagittal plane) without using crutches. Moreover, the stability region in double contact indicates that the operator may safely reposition both crutches during transfer phase in preparation of the next swing phase, without losing balance in the sagittal plane. The COM trajectory is also contained within the single contact BSB during the swing phase, only briefly exiting at the very end when the legs switch roles in preparation for the next transfer phase. The balance stability of the given crutched walking trials in relation with the calculated crutch-less stability boundaries indicates that a future balance controller could be designed such that the role of crutches in the sagittal plane balancing could be reduced in transfer phase and most of the swing phase. This enhanced sagittal plane balancing capability in single and double contacts is in part due to the full (including ankle) joint actuation in the sagittal plane in Mina v2. On the other hand, the role of crutches may still be relevant at the very end of the swing phase for the sagittal plane stability of Mina v2, and also at the swing and transfer phases for the lateral stability, since the system is unactuated and has restricted joint rotation in the frontal plane.

The calculated BSBs in the single and double contact configurations predict the contact-specific state space regions within which the combined human-exoskeleton system has the physical capability to maintain balance using the support of the leg(s) during any generic task, without ever altering the respective contacts. Therefore, these regions represent a contact-dependent system property that can be used as a reference for the design of task-specific controller domains in the state space, for which the contact-specific balanced and unbalanced regions are pre-computed (i.e., known *a priori*). As specific applications in robotic exoskeletons, the calculated balance stability regions would provide quantitative guidelines for the mechanical and control system design of robot-assisted locomotion. For instance, the BSBs can be used as reference maps for benchmarking human gait, improving the walking trajectory design at an early stage (before the trajectories' actual implementation and testing), and evaluating the role of crutches as balancing aids in multiple planes. The integration of the proposed balance stability criterion within novel human-robot interface technologies could provide the operator with quantitative feedbacks during training, hence providing assistance for the exploration of less conservative and more agile walking trajectories.

## Ethics statement

This study was carried out in accordance with the recommendations of The Institutional Review Boards (IRBs) of The Florida Institute for Human and Machine Cognition (IHMC) and New York University (NYU). The protocol was approved by the respective IRBs. All subjects gave written informed consent in accordance with the Declaration of Helsinki.

## Author contributions

CM developed the theory and the computational framework for balance stability, and led the modeling, coding, and analysis. WP developed the mechanical and actuation models of the equivalent DH system, and processed and analyzed the data. SA developed robot's mechanical model and the combined human-exoskeleton system. RG developed the control system and helped conduct the experiments. PN led the hardware design, performed all of the transformation and calculations for the actuator, and helped conduct the experiments. JK developed the theory, helped the modeling and analysis, and supervised the overall research. All the authors contributed to the discussion, writing, review, and editing of the article.

### Conflict of interest statement

The authors declare that the research was conducted in the absence of any commercial or financial relationships that could be construed as a potential conflict of interest.
